# Site-dependent shaping of field potential waveforms

**DOI:** 10.1093/cercor/bhac297

**Published:** 2022-08-16

**Authors:** Oscar Herreras, Daniel Torres, Gonzalo Martín-Vázquez, Sara Hernández-Recio, Víctor J López-Madrona, Nuria Benito, Valeri A Makarov, Julia Makarova

**Affiliations:** Department of Translational Neuroscience, Cajal Institute, CSIC, Av. Doctor Arce 37, Madrid 28002, Spain; Department of Translational Neuroscience, Cajal Institute, CSIC, Av. Doctor Arce 37, Madrid 28002, Spain; Department of Translational Neuroscience, Cajal Institute, CSIC, Av. Doctor Arce 37, Madrid 28002, Spain; Department of Translational Neuroscience, Cajal Institute, CSIC, Av. Doctor Arce 37, Madrid 28002, Spain; Department of Translational Neuroscience, Cajal Institute, CSIC, Av. Doctor Arce 37, Madrid 28002, Spain; Department of Translational Neuroscience, Cajal Institute, CSIC, Av. Doctor Arce 37, Madrid 28002, Spain; Department of Applied Mathematics, Institute for Interdisciplinary Mathematics, Universidad Complutense of Madrid, Av. Paraninfo s/n, Madrid 28040, Spain; Department of Translational Neuroscience, Cajal Institute, CSIC, Av. Doctor Arce 37, Madrid 28002, Spain; Department of Applied Mathematics, Institute for Interdisciplinary Mathematics, Universidad Complutense of Madrid, Av. Paraninfo s/n, Madrid 28040, Spain

**Keywords:** FP sources, LFPs, network oscillations, source mixing, volume conduction

## Abstract

The activity of neuron populations gives rise to field potentials (FPs) that extend beyond the sources. Their mixing in the volume dilutes the original temporal motifs in a site-dependent manner, a fact that has received little attention. And yet, it potentially rids of physiological significance the time-frequency parameters of individual waves (amplitude, phase, duration). This is most likely to happen when a single source or a local origin is erroneously assumed. Recent studies using spatial treatment of these signals and anatomically realistic modeling of neuron aggregates provide convincing evidence for the multisource origin and site-dependent blend of FPs. Thus, FPs generated in primary structures like the neocortex and hippocampus reach far and cross-contaminate each other but also, they add and even impose their temporal traits on distant regions. Furthermore, both structures house neurons that act as spatially distinct (but overlapped) FP sources whose activation is state, region, and time dependent, making the composition of so-called local FPs highly volatile and strongly site dependent. Since the spatial reach cannot be predicted without source geometry, it is important to assess whether waveforms and temporal motifs arise from a single source; otherwise, those from each of the co-active sources should be sought.

## Introduction

One highly pursued objective in brain physiology is to obtain a quantitative index of the activity of neuron populations with sufficient spatial and temporal definition to unravel the circuits involved in information processing during a specific behavior or to explore pathology. Such an ambitious objective seemed to be feasible when it was seen that brain waves could be associated with specific cognitive and behavioral manifestations (Berger 1929). However, a large body of clinical and neurophysiological literature uses these waves primarily as biomarkers whose anatomical ascription is, in most cases, vague or non-existent. One of the main obstacles to this achievement is the lack of spatial information of the participating neural current sources. As can be seen from contemporary monographs (Buzsáki et al. 2012; Pesaran et al. 2018), there is relatively little background information devoted to understanding how waveforms arise from the co-activation of multiple populations whose activities mix in the volume. Here, we argue that the lack of these data favors biased interpretation and prevents proper treatment of voltage fluctuations.

This position is essentially that presented by Elul (1971) in his instructive review half a century ago and little has changed since then (Bullock 1997; Herreras 2016). Despite the limited biophysical understanding of brain waves, as manifested through field potentials (FPs) and their surface counterpart, the EEG, they appear in the literature as a kind of magical biomarker that reveals the brains’ secrets by simply counting the number of waves per second (frequency bands). Waveform parameters obtained through time-frequency analyses (amplitude, duration, phase) at specific recording sites are widely used without a prior exploration of the contributing sources there (Cole and Voytek 2017; Wodeyar et al. 2021). But this is an insufficient treatment as it implicitly assumes that FPs reflect the temporal dynamics of a single source. Indeed, the literature rarely mentions that the time course of FPs may be useless as we do not know the number, the identity or the location of each source, and when they are activated, which applies for both irregular FPs and common network oscillations.

From the early studies by physics-oriented researchers, it was noticed that brain waves are a blend of potentials that originate from microscopic sources distributed in the volume, a complex mixture of heterogeneous neuron segments that inject current to the extracellular space and set space-varying electric fields that reach far from the sources. As this implies their mixing in the volume (Supplementary Video 1), it was anticipated how difficult the anatomical ascription of mesoscopic FPs would be (Lorente de Nó 1947a, 1947b; Woodbury 1960; Elul 1971; Nunez and Srinivasan 2006).

Progress in the last 10 years has finally begun to break the barriers towards achieving these objectives. Much improved multisite recordings, along with modern blind source separation (BSS) mathematical tools capable of separating the mixed sources (Bell and Sejnowski 1995; Makarov et al. 2010), and large-scale, anatomically detailed computational models of neurons and neuron aggregates (Varona et al. 2000; Lindén et al. 2011; Fernández-Ruiz et al. 2013; Reimann et al. 2013; Torres et al. 2019) allow the composite and spatial nature of FPs to be assessed and explored at a level that has been virtually inaccessible until recently. As such, we are now ready to tackle the main feature of FPs that compromises the reliability of waveforms and frequency bands, namely their site dependency.

We previously discussed some technical and theoretical issues that hinder the interpretation of FPs (Herreras 2016). Here, we focus on recent literature that supports the prevalence of a multisource nature of FPs in the brain, and the evidences for the main derived consequence, namely how the coactivation of different sources sets the time course of the associated potentials in a site-dependent manner, and we use simple numerical simulations to explain how this spatiotemporal blending gives rise to a large variety of waveforms and temporal motifs in different recording sites that cannot be unraveled by single-site recordings. Importantly, since site dependency is a property inherent to all mixtures, it affects the time course of LFPs whether or not they have contamination from distant sources, as they are themselves a mixture of nearby sources (Herreras et al. 2015). We first expose the general problem and provide the basics to help understand the importance of source geometry.

Earlier monographs provide an overview of issues related primarily to the temporal aspects of FPs (Buzsáki et al. 2012; Cole and Voytek 2017; Pesaran et al. 2018) and the effects of the electrical properties of the conducting medium (Malmivuo et al. 1995; Bédard and Destexhe 2009; Güllmar et al. 2010). For simplicity, we here follow the quasi-stationary approach in describing terms and relationships between physical variables, since source geometry of dipolar sources is limiting on the reach of FPs and supersedes non-ohmic effects (Lorente de Nó 1947a; discussed in Herreras 2016). For issues more related to spatial aspects, monographic and more specialized texts can be consulted (Woodbury 1960; Rall and Shepherd 1968; Elul 1971; Gloor 1985; López-Aguado et al. 2001; Nunez and Srinivasan 2006; Lindén et al. 2011; Herreras et al. 2015; Torres et al. 2019).

## From field potentials to microscopic sources and back: a complex but necessary journey

To facilitate the understanding, it seems pertinent to briefly mention the relationship between the geometry of a neural source of current and its associated potentials inside and outside the source, and also to dedicate a few words to the anatomical correlates of the sources themselves. These two important aspects have been notably upgraded in the late years (for a review see Herreras et al. 2015). The basic elements are schematically depicted in Box 1 (microscopic correlates of FP buildup) and Box 2 (anatomical conditions promoting the far reach of FPs), which jointly explain why the amplitude inside the source is variable and does not determine the spatial reach, an essential concept for understanding non-intuitive or seemingly discordant observations when potentials are used and interpreted without reference to their sources.

### Field potentials are a variant of the inverse problem of neurophysiology

There are multiple sources for FPs in the brain. Amongst the most relevant, the contribution of spikes, non-neuronal sources such as glia and even electro-diffusive forces have been discussed in recent monographs (Bédard et al. 2004; Buzsáki et al. 2012; Dreier et al. 2013; Lückl et al. 2018; Herreras and Makarova 2020). And yet, customary FPs are known to represent for the most part the sum of synaptic and intrinsic currents from multiple neurons (Elul 1971; Haberly and Shepherd 1973; Reimann et al. 2013) and hence, the spatial gradients of voltage are much smoother than for unitary spikes. Such gradients are inherent to the three-dimensional spread of potentials in a volume conductor from their sources (Supplementary Video 1) and are key to locate them in the volume (Herreras et al. 2015). The main practical handicap in this operation is that unlike some imaging techniques that can scan large portions or even the entire brain (e.g. functional magnetic resonance, diffusion tensor and functional ultrasound), electrodes pick up FPs at a single point in space and thus, they cannot provide information on the complex 3D shells of voltage nor how many sources have contributed.

Consequently, finding what information is retained in raw FPs is a thorny problem that is best addressed by turning to the ground truth of electric potentials, i.e. tracing the sources of current to find their location and geometry, with which anatomical ascription can be attempted. However, this is one of the fundamental problems in neurophysiology, known as the *inverse problem*, a well-known, century-long issue in the EEG community (Pascual-Marqui 1999; Nunez and Srinivasan 2006; Grech et al. 2008) that also affects intracranial recordings (Herreras et al. 2015). It states that a given FP series in a specific site can be raised by addition of potentials from countless combinations of—unknown—sources. Inside the brain, the addition of potentials from distant sources is typically considered and referred to as *volume contamination*, a term that is an inadequate nominalization of the superposition of fields generated by remote sources to others assumed to be near the electrode. In turn, the latter are usually referred to as LFPs, also an overly simplistic and misleading term as *local field* can be considered an oxymoron. The neuronal sources of current remain in place, but their associated potentials reach far out. For the sake of familiarity, we will retain the term LFP as a conceptual rather than factual entity to refer to potentials whose sources are presumed, or proven to be at the recording site.

**Fig. 1:**
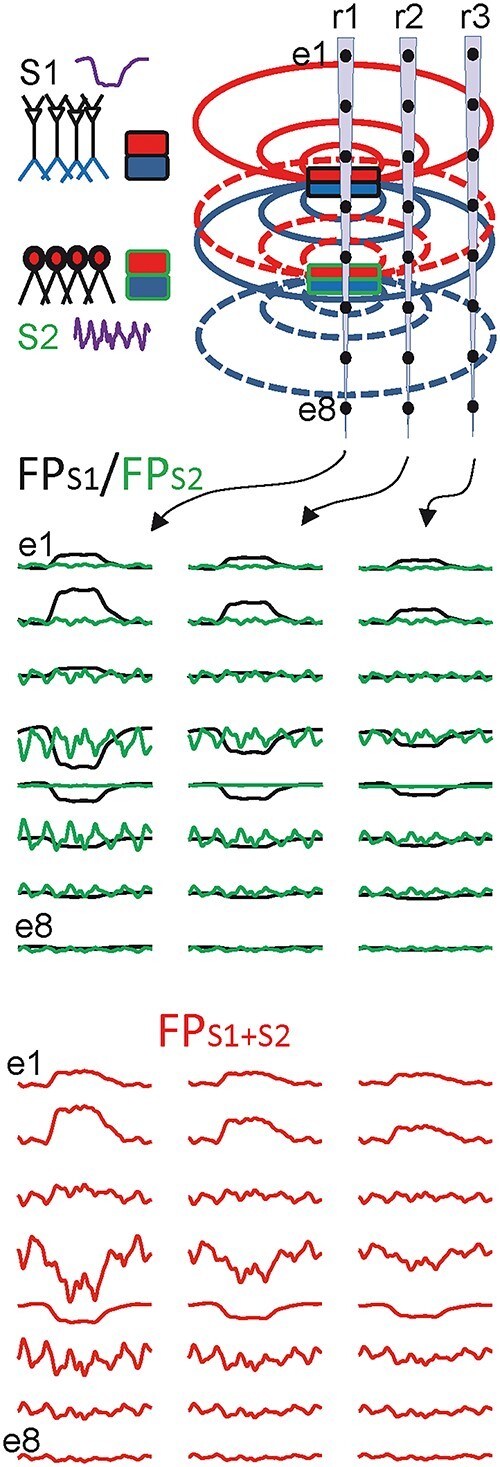
FPs with contributions from various sources add/subtract their temporal traits in a site-dependent manner. The upper panel depicts two dipolar sources (S1 and S2) and multisite recording arrays (r1–r3) covering a portion of the space nearby. The dipoles represent synaptic inputs to two different neuron populations with different dynamics (left). Middle: All sources produce field potentials (FPS1, FPS2) that decay in amplitude over a distance. Note that the temporal structure is maintained at all positions for the FPs of each source. Bottom: Since potentials add linearly in a volume conductor, when the two sources are co-activated (traces in red) the time course of the original sources is lost and the resulting FP pattern (FPS1 + S2) varies at different sites according to the relative distance to each of them. At some sites, it resembles one of the original sources but at others, the blending results in the disappearance of the traits identifying the original sources. Note that single-site recordings cannot provide the number, position or temporal features of the co-activating sources.

From a practical point of view, the more troubling features are (a) that the neuronal sources are dipolar (produce both positive and negative potentials in different zones), and (b) that potentials fall off with distance to the source. The biophysical consequences are illustrated in Fig. 1 through the spatial display of FPs elicited by two—dipolar—sources, either alone (black and green traces) or when co-activated (merged potentials in red). The coactivation of different sources always causes a distance-dependent superposition of their respective FPs in the volume, that is, each point in space contains a different mix of the same components but in a different ratio. Since each source has a time course, *the voltage waveforms thus composed will differ at different recording sites*. In some brain structures, FPs can vary quite dramatically within a few tens of microns (Elul 1962; Benito et al. 2014). Which site should be used? It can be anticipated that whether the co-active populations are distant (case of volume-conducted FPs) or close to each other (case of LFPs), the potentials added at any given recording position mask the original time traits of the sources of current, and they are likely to become electrical gibberish as the number increases. Indeed, experimental and model data reviewed below confirms that the intracranial recordings at any given site are insufficient to: (1) identify the site/s of origin of what is being recorded and (2) know which waves or parts of waves are generated by specific neuronal sources.

**Box 1:**
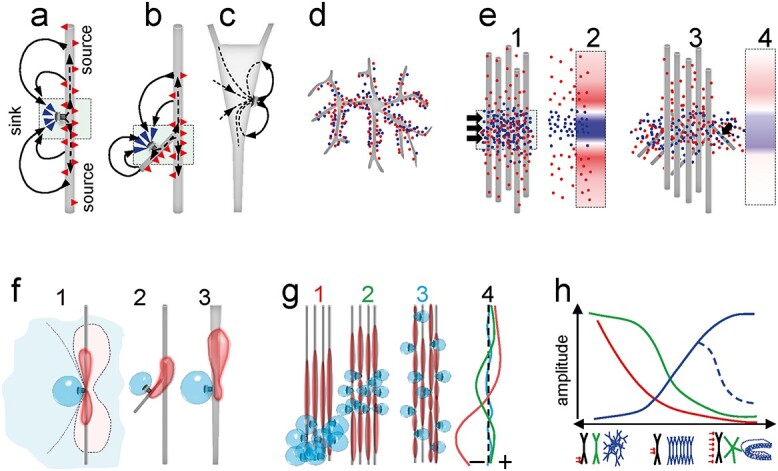
The neuron morphology and synapse location determine the amplitude of extracellular currents and potentials. During synaptic activation, electrical current traverses neuronal membranes twice (arrowheads), once as active (ionic) current through discrete points in the synapses, and once as return current to the extracellular space (ES). For illustration, panel “a” shows the currents during activation of an excitatory synapse. As the currents propagate inside, they leak out through the membrane capacitance (red arrowheads), so it will be the width of the dendrites and the branching pattern that determine how much leaks out close to the synapse, and therefore cancels with inward current there, and how much goes out far from it. In the diagram, the membrane patches where intense cancellation of inward and outward currents occurs is represented by squares. Zones where net current is inward or outward are called sinks or sources, respectively, and jointly form extracellular dipoles of current. These promote extracellular current to close the loop (curved arrows). Thus, knowing the 3D morphology of a neuron helps predicting if synaptic currents will manage to create source/sink dipoles in the ES and thus contribute to FPs. The cancellation of inward and outward currents in small volumes around the synaptic zone is minimized when synapses are located on a thick primary dendrite (a) so that returning currents separate maximally from active ones in the ES, but reduced when they are located on secondary oblique dendrites (b). Cancellation is much more pronounced in the cell body (c), because the large size and surface provides easy path for return currents at the same ES level of active currents. (d) The co-activation of multiple synapses in neurons with multipolar morphology also maximizes cancellation of inward and outward currents in cell-size volumes (dots represent microscopic sources and sinks). (e) Similarly, addition or cancellation between sinks and sources takes place when they arise from different neurons. Thus the buildup of multineuronal (mesoscopic) dipoles is facilitated in cases of homogeneous activation of spatially grouped excitatory synapses in aligned neurons (e1–2), and reduces as synapses are dispersed over the dendrite or when they are located in secondary oblique dendrites (e3–4). The net sources and sinks in the ES behave as layered blocks or sheets of current (red/blue bands) with spatially variable charge density and simplified geometry (e2, e4). This facilitates biophysical processing towards estimating the amplitude and extent of the associated FPs (bottom row). The spatial distribution of FPs can be well derived from the general geometry of a source/sink combination (thereafter referred to generically as source), and users should try to visualize them as concentric isopotential spheroids around it without a pre-set boundary (Supplementary Video 1). It is essential to remind that electric fields extend beyond the physical limits of the source, which makes the spatial distribution of FPs less intuitive (Box 2). Panels e and f show the partition of the ES into positive (redish) and negative regions (bluish) at an arbitrary isopotential level for the activation of a single (e) or a group of synapses (f). As for currents, the location of the synapse on a main (1) or a secondary oblique dendrite (2), or in a tapering dendrite (3) determines the relative size and location of the respective positive and negative zones and whether they coincide at a similar spatial level or they are separated. Larger FPs arise when activated synapses are clustered at distal (f1) compared to middle (f2) dendritic segments, which has great relevance for stratified synaptic pathways. However, disperse synapses (f3) lead to maximal mutual cancellation of positive and negative potentials (compare proportional spatial plots of FP amplitude at f4). The ability of different cytoarchitectonic elements to generate FPs are summarized in g; red: location and grouping of synapses; green: cell morphology; blue: arrangement of neurons. All of these factors are limiting, therefore, all must be favorable to allow mesoscopic buildup of FPs.

This is of utmost importance since FP waves or epochs are commonly characterized by the aforementioned time-dependent variables without drawing any inference about the number and/or location of the sources (Cole and Voytek 2017; Wodeyar et al. 2021). Unfortunately, such common quantifiers are only reliable when the potentials arise from a single current source (e.g. some evoked potentials). In such a case, the temporal pattern is space-invariant; i.e., the instantaneous voltage is proportional at all sites (e.g. Fig. 1, black traces). However, this is unlikely to occur in the brain where multiple populations are incessantly turned on and off.

### How much do FPs reflect the anatomical sources of current? The pathway-specific nature of mesoscopic FPs

A major drawback to the interpretation of FPs has been the lack of a simple correspondence of mesoscopic FPs with their microscopic sources of current, which are mostly neuronal membranes. Understanding how extracellular currents are contiguous with intracellular ones is important because the inward and outward flow across different parts of the membrane establishes a dipolar nature for extracellular currents, the cancellation of which can be massive when volume averaged (Box 1). A snapshot of these currents would provide the 3D geometry of the zones where, in average, neuron membranes act as sources or sinks of current in the ES. While it is clear that the membrane patches that act as a sink or a source during single synapse activation have a different geometry, the strong cancellation of microscopic currents during collective synaptic activation causes that the geometry of mesoscopic sources and sinks cannot be anticipated without detailed anatomical information. This is perhaps the most underappreciated principle whose neglect is at the root of a pervasive simplistic treatment of brain potentials. It has been long known that the electrical behavior of the neuronal mass that gives rise to FPs is not a simple transfer function of the activity in the individual neurons (Freeman 1975), or a weighted estimation of the number of activated synapses (Elul 1971). One cannot easily determine what is lost and what remains in this process, since the 3D cloud of microscopic sources and sinks is jointly defined by cytoarchitectonic factors at three different spatial scales, the subcellular synaptic territory of synaptic pathways, the morphology of the neurons, and the arrangement of the population (López-Aguado et al. 2002). Consequently, it should be established empirically on a case-by-case basis. Definitely, we are a long way from constructing a complete picture of micro-to-mesoscopic scaling (Hales and Pockett 2014). Yet, improved computational capabilities have become of great help for describing the general principles that relate each of these anatomical levels to the extracellular FP gradients (Lindén et al. 2011; Makarova et al. 2011; Fernández-Ruiz et al. 2013; Torres et al. 2019).

Box 1 illustrates the relevance of the location of individual synapses along the dendritic arbor and the effect of varying distributions of groups of synapses for the buildup of FPs inside the source population. The most favorable synaptic grouping to raise measurable FPs occurs during the natural activation of homogeneous populations by a common pathway (i.e. cell assemblies), which provides the necessary synchrony and spatial clustering of many homologous synaptic inputs. Synchronization of afferent fibers is obviously a necessary requirement, but it is the merging of their synaptic territories that is translated post-synaptically into a spatial module of coherent mesoscopic FP activity (Benito et al. 2014; Rogers et al. 2019). This makes most FPs a conglomerate of pathway-specific potentials (Herreras et al. 2015). Thus, we use the term *source of an FP* to refer to *the extracellular currents produced by a specific neuron population upon synaptic input from a given pathway,* also referred to as a FP generator.

The pathway specificity of FP sources is a key concept that provides a mesoscopic platform from which numerous issues related to the spatiotemporal diversity of FPs can be better understood. Abundant literature on evoked FPs has shown characteristic voltage profiles that are specific for many pathway-to-population combinations (Andersen et al. 1971; Mitzdorf and Singer 1978; Di et al. 1990; Leung et al. 1995; Kolb et al. 1997; Canning et al. 2000; Martín-Vázquez et al. 2016). These have been confirmed by studies using spike-triggered FPs from single afferent neurons (Fernández-Ruiz, Makarov, Benito, et al. 2012a; Fernández-Ruiz, Makarov, Herreras 2012b; Bereshpolova et al. 2019). Recognizing the sources of spontaneous FPs is, however, extremely difficult (Supplementary Video 1), but the pathway-specific basis has finally been confirmed by studies in the last decade demonstrating that ever changing spatial profiles of spontaneous FPs arise from the varying combination of a few spatially coherent contributions whose profile could be matched to those of evoked potentials in individual synaptic pathways (Korovaichuk et al. 2010; Benito et al. 2014; Herreras et al. 2015).

This anatomical basis for mesoscopic FPs helps to better understand why a single neuron (or a homogeneous population) harbors different source geometries depending on the portion of its dendritic arborization that is activated by one or another pathway. Supplementary Video 2 dynamically recreates this important concept from calculations performed on a morphologically realistic aggregate of the CA1 pyramidal population of the hippocampus (Martín-Vázquez et al. 2016). Experiments in the past decade have shown that the number of pathway-specific FP generators in large structures as the cortex and hippocampus is much smaller than that of anatomical pathways (Benito et al. 2014; Torres et al. 2019), supporting the theoretical expectation that most pathways do not contribute to FPs due to their unsuitable geometry (Lorente de Nó 1947b), also confirmed by detailed anatomical computations (Lindén et al. 2011; Makarova et al. 2011; Martín-Vázquez et al. 2016; Torres et al. 2019).

### Understanding FP waveforms through pathway-specific dynamics

Any FP time series is a succession of waves and these may be quite similar (rhythmic oscillations) or a relentless sequence of complex waveforms (irregular activity) (Bullock et al. 2003). How should we interpret these waveforms? Is a wave generated by a single source? How can we know if it results from multiple sources? The pathway-specific nature of FPs not only helps the anatomical interpretation, it also implies that the FP waveforms are tightly dependent on the activation dynamics in each pathway (Makarova et al. 2011). Note that asynchronous input does not permit the temporal summation of currents as the microscopic fields do not add to attain measurable amplitude (Elul 1971).

**Fig. 2:**
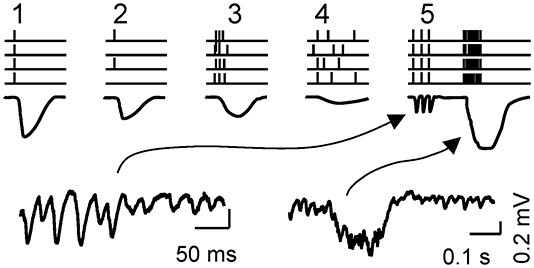
Different dynamics of a single upstream population produce different FP waveforms. Although variable FP waveforms are expected from the mix of sources, a single source can also produce varying waveforms. The diagrams at the top show the shaping of waveforms by varying the timing of the spikes in the afferent neurons (vertical strokes): 1–2, full synchrony; 3–4, increasing the temporal dispersion. Note that the amplitude depends on both the synchrony and the number of elementary inputs (black traces). Only full synchrony preserves the waveform of the elementary inputs. 5, different upstream spiking regimes give rise to very diverse FP motifs. The lower traces correspond to Schaffer-specific FPs in a real experiment, which may produce gamma oscillations and sharp-wave events.

The simplest case of FP waves is that produced by the summation of post-synaptic currents during synchronous activation of the fibers in the same pathway (i.e. single-sourced) (Fig. 2). In this case, a smooth waveform closely matches that of the elementary synaptic event. This is the case of customary evoked potentials (Mitzdorf and Singer 1978; Di et al. 1990; Arieli et al. 1996; Canning et al. 2000), although these generally arise in conditions of strong spatiotemporal clustering and homogeneity that are not applicable to spontaneous FPs. Such conservative micro-to-mesoscopic up-scaling of waveforms has been experimentally reported in some cases when the activity of individual sources can be separated to unravel reliable time courses (Herreras et al. 2015). For instance, our team managed to mathematically extract the Schaffer-specific FPs in the hippocampal CA1 sub-field (Fernández-Ruiz, Makarov, Benito, et al. 2012a), which displayed bouts of gamma waves. We concluded that each wave corresponded to the synaptic envelope produced in the CA1 by highly synchronous input from a CA3 pyramidal cell assembly. Thus, gamma waves exhibit a substantial variability in amplitude (Fig. 2, bottom left), yet this is less marked in terms of duration, and of the rising and falling times (Benito et al. 2016), which is best explained by the size and internal synchrony of the afferent assembly (Kopell and LeMasson 1994). Interestingly, the same synaptic source gives rise to much longer and larger FP events, known as sharp-waves (Fig. 2, bottom right; Ylinen et al. 1995; Fernández-Ruiz, Makarov, Benito, et al. 2012a; Benito et al. 2016). Thus, a varying temporal jitter of afferent neurons leads to the production of different FP waveforms by the target population, also contributing to different frequency bands. These findings demonstrate empirically that a single source can provide a variety of FP waveforms. This might be expected if we consider the wide dynamic ranges of spike firing in most neuron populations and the multiple cell subtypes firing at similar frequencies (Ranck 1973; Clawson et al. 2018).

### Relationship between source geometry and the reach of FPs

In order for a brain source to influence the time course of others, its associated potentials must invade the territories of others. This is a more common situation than is generally thought. Actually, it is the common case in LFPs. FP signals are customarily marked as local (LFPs) or remote on the assumption that the sources are close to or far from the electrode, respectively. But, is the closest source always that which governs the time course of a recording? Some studies that explored the spatial spread of FPs endorsed the view that they are mostly local (Berens et al. 2008; Katzner et al. 2009; Liu et al. 2015). However, in these studies, the sources were small and are by no means representative of the extremely variable size and changing geometry of the sources whose potentials sprawl the brain. The localistic interpretation of recordings is obviously at odds with the physical principle of an electric field reaching anywhere and should not be taken for granted (see Supplementary Video 1).

The reach of an FP from the source depends on three factors: the source geometry, the degree of activation, and the electric properties of the conducting medium. The latter two have been widely covered in the literature (Li et al. 1968; Elul 1971; López-Aguado et al. 2001; Herreras 2016), but less so the geometry of the source, which is the most important because it is a limiting factor. The general rules have been known for some time (Lorente de Nó 1947a, 1947b; Woodbury 1960; Klee and Rall 1977; López-Aguado et al. 2002), yet a detailed analysis of specific structures has only been performed in a few cases using detailed computational models with realistic neuron morphology and arrangement (Makarova et al. 2011; Fernández-Ruiz et al. 2013; Reimann et al. 2013; Martín-Vázquez et al. 2016; Torres et al. 2019; Bertone-Cueto et al. 2020). Other non-geometrical approaches have also been deployed that need for experimental assessment (Ho et al. 2012; Telenczuk et al. 2020). In Box 2, some of the geometrical aspects promoting large FPs inside thesource and the far reach are depicted schematically. The cytoarchitecture of the population (orderly vs. glomerular), the morphology of the neurons (axial vs. multipolar) and the location of the synaptic inputs (clustered vs. scattered) are the main aspects to be borne in mind. Importantly, all three spatial scales are limiting, so they all need be met to envisage the FP-generating capability of a brain structure (Nunez and Srinivasan 2010). Many combinations of these three factors promote FPs of large amplitude inside the source. However, only some favor the far reach, such as the activation of pathways making synaptic contact in distal portions of well aligned neurons, or the folding of layered populations (Fernández-Ruiz et al. 2013; Martín-Vázquez et al. 2016).

**Box 2:**
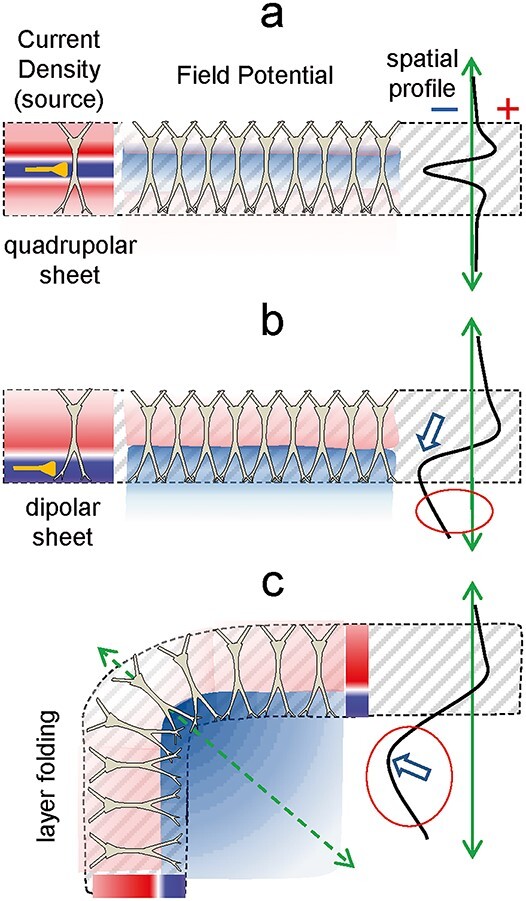
Architectonic factors promoting the far reach of FPs. One might expect an FP to fall towards zero from the source, and the rate of decay to be proportional to the amplitude at the origin. However, neither of these is correct. The amplitude of the FPs within the limits of the source population does not define whether or to what extent they will spread to other structures. This is a transcript of Coulomb’s law, according to which there are no spatial limits for the electric field generated by charged particles. Therefore, since the sources and the sinks that form an extracellular dipole are neither regular nor uniform, the strength and rate of decay of the combined field they create varies in different directions depending on how each is located relative to each other. The 3D geometry and density can be very complex. Some general principles can be derived from the activation of orderly populations, which can help in more complex cases. In palisade-arranged populations of neurons (e.g. cortex and hippocampus), in which principal cells meet the criteria for generating large FPs (Box 1), it is the location of the afferent pathway in the dendritic tree which determines whether it also extends far in volume. Thus, inputs to distal dendrites tend to generate mesoscopic dipoles (dipole sheets) that promote far reaching of associated FPs away from source neurons, whereas inputs to middle dendritic segments lead to a configuration of quadrupole (sandwich-shaped), whose FPs tend to “stay” local (compare diagrams a and b). Even though the amplitude of the LFPs may be equivalent for the two input locations, the rate of decay is much more pronounced when the active (synaptic) layer that accumulates a strong sink is surrounded by sources from both sides (see spatial profiles on the right), as if it were shielded. The red circle in b indicates the far reach of the FPs away from the source population. Another common architectural configuration in the brain is that of folded layers of cells. The curvatures thus formed cause remarkable effects when combined with activation in distal dendritic segments (c). Thus, even when individual cells produce the same configuration of currents, the amplitude of FPs on the concave side of such cell arrangement exceeds several times the achieved in the synaptic layer itself, while it decreases somewhat on the convex side. This can be understood considering that, on average, the individual sinks are much closer to any site in the concave region than they would be in a parallel arrangement. This also calls for caution when using the maximum voltage (white arrows) as a marker of source location: it may not even be close.

Assuming none of the mentioned anatomical limiting features is at play, size can be considered the fourth factor determining the reach of FPs. For instance, extended sources produce FPs that decay over distance at much lower rate (i.e. reach further) than those produced by small sources, such as some of those generated in the cortex or hippocampus (for an extended analysis and discussion see Torres et al. 2019). Yet, there are abundant examples in the brain that size is subordinate to the other geometrical factors. Some large structures like the striatum are poor generators of FPs due to their inadequate neuron morphology (e.g. multipolar; Martín-Vázquez et al. 2016; Bertone-Cueto et al. 2019). Thus, FP activity recorded in these regions is mostly originated in distant sources (Lalla et al. 2017; Parabucki and Lampl 2017; Torres et al. 2019).

## FP waveforms originating from multiple sources vary at different sites

The FP literature focuses primarily on visually recognizable time patterns, whether they are rhythmic oscillations or stereotyped wave sequences. Amongst others, sharp waves, ripples, spindles, delta waves, dentate spikes and a variety of rhythmic oscillations are often taken as operational blocks of activity in specific neural circuits and structures, and they are widely used as markers of behavioral/cognitive tasks. The overall stability of these FP events across experiments gives them functional identity and it reflects their origin in specific network segments or neuron populations in each case. However, the cellular bases (the pathway and population making up the source) of typical FP events are not fully clarified (Buzsáki et al. 2012; Herreras 2016). Thus, there is ample room for refinement since earlier works have addressed them without considering the spatial dimension and/or their multisource nature. It has been shown that many of these that, tacitly or not, were previously considered single-source events are actually compound waves from two or more sources, with characteristic site-dependent waveforms. These include customary theta and gamma oscillations in the hippocampus (López-Madrona et al. 2020) and gamma, delta or alpha waves in the cortex (Ortuño et al. 2019; Torres et al. 2019). Their quantification and statistics are generally obtained from time series analysis (e.g. Fourier approaches) of single-site recordings, which cannot unravel the different sources and their relative contributions. As we shall appreciate below such parameters will rarely coincide with those of the contributing sources and may thus become meaningless.

Below we discuss how factors such as the size, shape, location and distance between the sources and the electrode define the spatial variations of the waveform in the vicinity of the electrode. We first expose the principles using idealized FP waveforms originating from two nearby sources and then the experimental cases will be reviewed.

### Site-dependent waveforms upon mixing of co-localized sources: LFPs

**Fig. 3:**
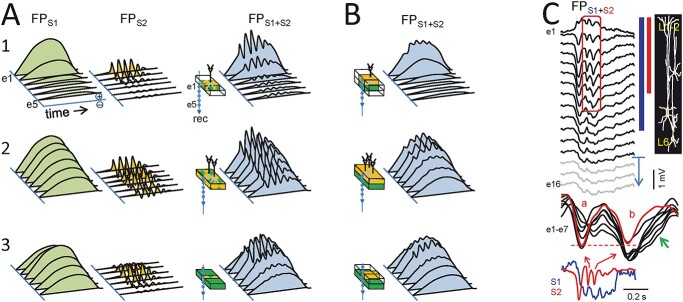
Site-dependent waveforms in LFPs are caused by the mixing of partially co-localized sources. (A and B). The diagrams show schematically the effect of source geometry on the FP waveforms along a recording track (e1–e5) covering positions in and near the sources. These are depicted as colored blocks on neuron dummies, and the extent denotes the size of the population and dendritic domain that is coherently activated. Colored waveforms are generated by each of the two sources alone (green and orange) or when co-activated (blue). Different dynamics for the sources have been chosen to facilitate visual discrimination of temporal traits: In (A), the sources arise from inputs in the same dendritic domain; and in (B), they are in adjacent domains of the same population. Rows 1 and 2 show the waveforms from two sources of identical extension, and in row 3, they have different extensions. Co-activation always results in site-dependent waveforms, although different sets are obtained for different geometrical configurations and dendritic position of the sources. For simplicity, only one polarity is used. (C) Example of co-localized sources rising site-dependent LFPs in real experiments. The laminar profile corresponds to a slow cortical wave (S1) with superimposed spindle-like oscillations (S2). The two sources extend through different portions of the cortical column (colored bars to the right). Since the slow wave also has fast fluctuations (see the time course in the blue trace at the bottom), their mixing with waves produced by the spindle source that have different phase and a varying power along the cortical column renders strong site-dependent waveforms. The characteristic signature of multisource contribution is the dissimilar profile exhibited by contiguous waves. The superposition of FP waves in sites e1–e7 reveals large variations in the amplitude, start/end-times and total duration (green arrow), and peak phase in different sites and from wave to wave.

We first consider how the size of co-localized sources affects the waveforms of potentials recorded locally and at short distances away from them (Fig. 3). This case concerns to ideal LFPs, i.e. those without significant contribution from distant sources. The simulated potentials correspond to the activation of two synaptic pathways targeting a population at the same distal dendritic position (Fig. 3A), or one of them making contact in the middle dendritic domains (Fig. 3B). In the first case (Fig. 3A, rows 1 and 2), both the single-source FPs (FPS1 or FPS2) and the merged ones (FPS1 + S2) decay from a maximum inside the source outwards at a similar rate, and this depends on the size of the sub-population activated. The FP waveform is identical at all recording points except that it is down-scaled by distance. However, when the sources belong to sub-populations of different size (i.e. the respective pathways activate neurons over territories of different size: Fig. 3A, row 3) the different rate of decay promotes site-specific weighting. Hence, the composite waveforms (in blue) vary depending on whether the recording position is in or close to the sources. It should be noted that at greater distances the waveform gradually stabilizes as the reduced gradients cause the relative contribution to equalize.

When the synaptic pathways target different dendritic domains (Fig. 3B), the compound FP waveform will vary greatly with recording location, and the time course will be dominated by the source making contact with distal dendrites due to the stronger dipolar moment (lower rate of decay). Note that mid-dendritic and distal dendritic inputs raise potentials with a rather different rate of decay, the former adopting quadrupole configuration (Box 1) that generates smaller and less far-reaching potentials.

It can be easily inferred that any combination of the geometric factors for a pair or more coactive sources will produce a site-dependent blending of the associated potentials. Note that since the extracellular space is essentially resistive, the frequency of the waves is irrelevant to define how far each spreads from its source (Herreras 2016; but see Bédard et al. 2004). In the example illustrated in Fig. 3A and B, we chose different dominant frequency for each of the sources to facilitate visual examination of changes in the composite waveform. Ideally, these two activities could be separated by bandpass filtering. Common cases found in the literature using such procedure are the entrained gamma and theta oscillations in the hippocampus, or the gamma or alpha oscillations superimposed on slow (delta) cortical waves (Fig. 3C) . Importantly, in practice, slow waves are rarely smooth and their ongoing—faster—fluctuations are closer in duration to faster oscillations from other sources that impede a safe visual discrimination of each source’s time course (green arrow in Fig. 3C). As a result, the amplitude, duration (frequency), phase and start/end times vary in different nearby sites and the changes are not stationary as they can vary from wave to wave (compare site dependence of waves a and b).

Therefore, although bandpass filtering can be effective in removing waveforms of unwanted duration, it does not guarantee that the remaining waves belong to a single source and will always leave “pieces” of all co-active sources. That means that filtered waveforms still contain multisource contribution and hence their parameters are unreliable. This problem is aggravated when co-located sources have a very similar frequency, in which raw FPs are not even suspected to arise from multiple sources (see below).

### Site-dependent waveforms upon mixing of potentials from far and near sources: “volume contamination”

**Fig. 4:**
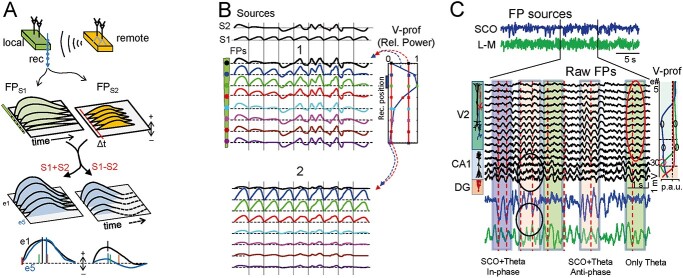
Site-dependent waveforms caused by the mixing of potentials from far and nearby sources (volume contamination). **(**A) Schematic analysis of the FP obtained near a source (S1, local) to which site potentials also arrive from another distant (contaminating) source (S2). Note the fast decay of the FPs for the local source (green) and the constant amplitude of the remotely generated ones (orange). Similar waveforms have been chosen for the two sources and a slight delay (Δ*t*) has been allowed to indicate independence. The effect of the remote source is to add waveforms with a constant voltage to all sites near the local source, whose waves show a steep decay. When the local and remote sources have the same polarity, the blend of FPs (S1 plus S2, in blue) shows a slower decay rate and changes in waveform parameters at different sites (samples at bottom are normalized for comparison). When they have opposite polarity (S1 minus S2), the site-dependent waveform differences are much more marked. (B) Numerous site-dependent waveform changes can be observed when similar local waves blend to remote ones of varying amplitude, polarity, phase, duration or start time. The degree of site-dependent alterations is more marked when the remote FPs are stronger (1) and less perceptible when they are weaker (2) (relative power indicated in the respective spatial curves to the right). (C) Experimental example illustrating one such combination of near and far theta sources in the cortex (V2) and hippocampus (CA1). The multisite recording covers both regions, so which is close and which is local depends on the site inspected. Both structures can show genuine theta generators that coincide at certain epochs. The cortical FP generator is called slow cortical oscillation (SCO) as it normally shows such waves. Independent component analysis was used to get the correct time course on each (blue and green traces at top), which helped reveal the correct wave parameters at the sources. The colored boxes indicate epochs when the cortical (SCO) and hippocampal theta waves have different phases. Theta FP propagation in each territory produces a variety of site-dependent wave parameter distortions, such as amplitude modulation, dephasing, and even annihilation, affecting specific sites according to the strength of each generator in a given position (check, for example, the sites marked in red or blue ovals in the voltage profiles to the right). (C) Modified from Torres et al. 2019.

Field potentials merged from the blending of a local and a remote source represent a similar problem as that of two nearby sources except for the fact that far sources contribute about the same voltage in different sites near the local source (Torres et al. 2019). A site-dependent proportion of contributing sources is still maintained by the varying contribution of those nearby. The distortion of the merged waveform with respect to the local one depends on how similar is this compared to the distant one and their timing. Figure 4A illustrates a simple case through the mixing of potentials from separated sources with similar waveforms while recording at different sites near one of them (local). The main distortion affects the rates of voltage raise and decay. Also the peak time deviates notably from either of the original sources. The differences become drastic when the local and remote sources are not timed or if they have different polarity (compare S1 plus or minus S2), in which case the local waveforms become unrecognizable in the merged FPs.

It is intuitive that the spatial blending of waves of different wavelength, polarity, shape, phase, etc. produces an extraordinary variety of composite waveforms whose parameters may differ little or dramatically from the original sources. This depends on the relative power of the local and remote potentials at every site, which can be read from the respective spatial curves. In Fig. 4B, we consider a selection of the possible interactions in a numeric blend of an oscillatory epoch in the local FP (S1, LFP) made up of a rhythmic series of identical waves and potentials from a distant source of uneven waveforms, some similar and others less so (S2, remote FPs). When the distant contribution maintains high power at the recording position (Fig. 4B, 1), the merged waveforms are heavily site-dependent and many different distortions affect all the wave parameters, even the rhythmic sequence is blurred (waves 4–9). The severity of the distortions, and the areas where they occur, depend on the absolute value of the waves from each source at each position (see V-profiles). When distant contributions are weaker (Fig. 4B, 2), the distortions are less marked near the local source but more so in the regions further away where both become weak. One can easily figure out a similar outcome when the remote waves are equal but the local ones are not.

### Local and remote theta waves in the cortex and hippocampus: mutual distortion, spurious correlations, and other phenomena

The composite nature of FPs generated from near and far sources with similar waveforms can easily go unnoticed, yet they are very common. We can start with the theta activity, a network oscillation that may appear simultaneously in the hippocampus and the prefrontal cortex, and that exhibits behaviorally modulated coherence in certain epochs (Jones and Wilson 2005). Such momentary coupling is customarily interpreted as one structure driving the other through connecting pathways, although there may be alternative explanations. For instance, recent data obtained through spatial discrimination techniques applied to cortical FP profiles in anesthetized animals show that cortical theta waves belong to the same cell generator as delta waves, i.e. they are “short” up/down episodes that are repeated with a slightly higher frequency (Torres et al. 2019). Hence, some theta oscillations are set in the cortex by the thalamo-cortical system and in the hippocampus by the septo-hippocampal one. Although these two systems may engage during specific tasks (Tavares and Tort 2022), there may be alternative anatomical substrates that also happen to display FPs in the theta frequency (Nguyen Chi et al. 2016; Tort et al. 2018).

Using the dominant frequency of FPs to determine whether two networks are functionally engaged in short epochs or if they are coupled by chance may require extensive investigation and cumbersome statistical analysis. Having each one’s uncontaminated time course at hand greatly simplifies this task. For instance, none of the mentioned theta sources (prefrontal cortex and hippocampus) spread significant FPs through the volume to the other region, but they may effectively undergo exchange with nearer areas like the visual cortex. There, hippocampal theta waves of remote origin have been revealed by pharmacological blockade of local cortical activity, and conversely, cortical theta waves enter the hippocampus at high power (Munro Krull et al. 2019; Torres et al. 2019). Mutual distortions of theta waves between these two regions show striking site-dependent distortions of the amplitude and phase that are dependent on whether the cortical and hippocampal theta activities are in phase, out of phase or in antiphase (Fig. 4C). Even some waves may cancel completely at some sites but are still present at nearby ones, and this may be true for some waves but differ a few waves later. Such phenomena reveal the inadequacy of just selecting a reference site for phase assessment: source disentanglement is necessary. For instance, an inconsistent phase-lock of theta waves in two structures is poorly compatible with the theta activity in one driving the other. Naturally, since there may be genuine hippocampal or septum-driven cortical theta oscillations, identifying the sources (path and target cells) is undoubtedly the safer approach to rule out many possible confounders caused by insufficient or inadequate exploration of the FP spatial profiles (Mouchati et al. 2020).

There are other FP generators in the cortex and hippocampus that contain waves of similar frequency, and that interact in distant sites. Thus, slow waves (0.5–1.5 Hz) can also be generated in the hippocampus, for example by the entorhinal cortex input to the DG (Wolansky et al. 2006; Benito et al. 2014; Lockmann et al. 2016). Notably, while the cortex exports slow FP waves to the hippocampus and they contaminate FPs there, we rarely found the opposite to occur, seemingly because the FPs from DG sources spread preferentially towards the concave side due to the effective cancellation of opposing dipoles in the outer zones (Fernández-Ruiz et al. 2013). Thus, volume contamination of FPs from one structure to another is a pathway-specific phenomenon that affects some waves but not others, in one direction but not necessarily in the other.

### Hippocampal theta waves are shaped by multiple theta sources

The estimation of waveform parameters of hippocampal theta waves enters a higher level of complexity if we take into account that in addition to possible distortion by remote sources, each one may arise as a combination of multiple nearby sources. The existence of multiple intrahippocampal theta generators is long known (Brankačk et al. 1993; Kocsis et al. 1994; Montgomery et al. 2009; Pignatelli et al. 2012). This fact has, however, received little attention and theta wave parameters are typically quantified as if they arise from a single source. Recently, spatial discrimination techniques revealed three anatomically different sources of intrahippocampal theta oscillators with phase differences amongst them (López-Madrona et al. 2020). The substantial temporal overlap and the proximity of the respective sources indicate that all three sculpt the amplitude and phase of theta waves in raw FPs, albeit with variable power at different sites. This has important implications as one or more theta sources may be transiently restricted or switched off, which shall lead to moderate changes of waveform and amplitude at particular sites that go unnoticed in single site recordings and may be erroneously interpreted as intrinsic variability of a single theta generator. The chances are therefore high that theta epochs contain numerous waves with varying contribution from each theta source, causing their correlation with spikes to be spurious or biased.

### Melting pots of remote and nearby gamma sources

**Fig. 5:**
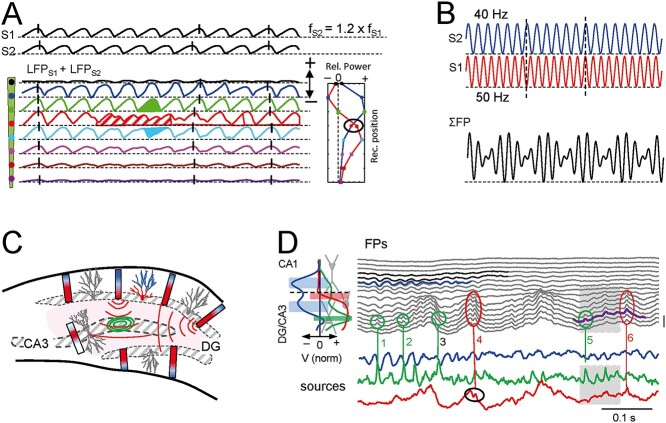
Site-dependent waveform alterations and other phenomena arising from mixing nearby oscillatory sources of similar frequency. Such cases are common for gamma sources in the cortex and hippocampus. (A) Theoretical analysis (numerical simulations). Note the different distortions that occur at different recording times and positions, including frequency doubling (red shadow epoch), polarity reversal (green and cyan waves), and inconsistent phase shift of waves over time and space (vertical strokes). In (B), the result at a single position is described over a longer period, allowing the phase shift to cycle. A curious amplitude modulation appears as if there are two sources with nested frequencies (the slower one is spurious) that resembles theta-gamma coupling. (C and D) Experimental example of gamma sources mixing in the DG. GCs behave as radially oriented dipoles when activated from the entorhinal cortex at a gamma frequency (C). Gamma oscillations blend in the Hilus (red curved lines), invading area CA3, which also generates gamma oscillations. The latter have a limited spread (green ovals) because the synaptic locus causes these cells to act as quadrupoles. In (D), laminar FPs are shown (black traces) containing multiple sources of gamma activity spanning the CA1 and DG/CA3 regions. The time course of the untangled sources is shown in colored traces. These allow the waves arising from a specific source or a mixture of different sources to be identified and the shape and phase of each to be determined. For example, waves 1, 2 and 6 belong to the CA3 source, while waves 4 and 6 arise from a DG source. It also helps to understand site-specific waveforms, such as wave 3 that has distorted waveform as it rides on top of a slower larger wave from the GD source. (C and D) Modified from Benito et al. (2014).

A better known example of multisource oscillatory FPs is gamma oscillations. These have been studied particularly in the cortex and hippocampus where multiple gamma sources overlap strongly but incompletely across layers (Martín-Vázquez et al. 2013; Benito et al. 2014; Ortuño et al. 2019). Oscillatory gamma sources may have slightly different frequencies (high and low gamma), and they may be phase-shifted and de-coupled (Zheng et al. 2015). Realistic model computations and numerical simulations show that such mixing leads to a number of spatio-temporal phenomena that are hardly intuitive and cause the temporal dynamics of the merged FPs to deviate markedly from that of the sources (Martín-Vázquez et al. 2013). Figure 5A and B shows some of these. For instance, cumulative phase offsetting will eventually match peaks in one and valleys in the other source, variably promoting addition or subtraction over time in some strata but not in others. The strong spatial overlap of the respective dipoles makes waves to add with a different relative power at each site. One may even appreciate sites in which some waves appear to show opposite polarities (e.g. green and cyan waves), but polarity matches a few waves later. Amongst relevant temporal distortions the addition of waves from sources with an appropriate phase offset may give way to merged FPs in which the frequency is doubled (red shaded period) in some sites but not in others. Notably, the phase of waveforms along a recording track turns inconsistent over time (vertical dashes). Importantly, spurious amplitude modulations of the successive waves commonly result from such mixings (Fig. 5B). Clearly, the parameters of FP waves are site-dependent and cannot be used as a proxy of those in the sources. Such variety of spatiotemporal phenomena is to be expected when the mixing sources are independent from each other, and it would serve the experimenter to tag the FPs as multisource and potentially unreliable. This will not be that conspicuous when the oscillatory sources are entrained (e.g. they keep a certain phase lag), in which case the multisource nature may go unnoticed.

Similar phenomena are commonly observed in experimental gamma oscillations. For instance, it is common to find phase transitions across the spatial profiles when exploring gamma oscillations in laminated structures (Orczyk et al. 2021). This cannot be directly interpreted from the raw FPs as they may be generated in multiple ways, such as from the spatiotemporal overlap of FPs elicited by two time-shifted gamma sources in different layers (Fig. 5A).

Spatial discrimination techniques have identified a significant number of excitatory and inhibitory oscillatory gamma sources whose spatial profiles overlap considerably, both in the hippocampus and the cortex (Makarov et al. 2010; Benito et al. 2016; Ortuño et al. 2019; Torres et al. 2019). Some of them spread beyond the structure of origin and may contaminate FPs in adjacent regions. Others remain circumscribed to the area in which they originate, where they can still mix with others. A true melting pot of gamma sources occurs in the CA1-CA3-DG regions (Fig. 5C and D). The DG exhibits up to three different oscillatory gamma sources arising from activation in as many synaptic pathways making contact in granule cells (Benito et al. 2014). Because the granule cell layer folding, the potentials grow abnormally large in the concave portion, the hilus, where they mix and become undistinguishable from one another. Further, an additional gamma source has been reported to arise from the cell body layer of the CA3–CA4 pyramidal neurons that populate the hilus (Benito et al. 2016; Martín-Vázquez et al. 2016). The FPs recorded in this area (Fig. 5D, gray lines) show series of apparently analogous gamma waves, although it is impossible to know which wave comes from local currents in CA3 pyramidal cells or arrives through the volume from the granule cells unless the spatial profile is available. For instance, single-point recording would not discriminate the different origin of waves 5 and 6 in the fragment outlined in purple. Even when multisite recordings are available, the part contributed by each cannot be determined when the two are activated together. Indeed, the variable phase-locking between waves elicited by the two pathways and the distinct amplitude of those originated remotely leads to a complex collection of site-dependent waveforms for successive waves (as in Fig. 5A).

A number of additional examples of gamma FPs blended from several nearby synaptic sources have been reported in the cortex and hippocampus (Benito et al. 2014, 2016; Ortuño et al. 2019). Some sources are located head-to-head in different populations, such as those at each side of the hippocampal fissure. More frequently, different pathways enter the same neuron population in adjacent somato-dendritic domains (e.g. the MPP and LPP inputs to granule cells). In the multilayered cortex, different synaptic inputs activate overlapping domains of the heavily intertwined dendritic arbors of different pyramidal cell classes. In addition, a component gamma oscillation is persistently found in the cortex whose remote origin is yet to be determined. Disentangling all these gamma sources is very demanding and even with spatial discrimination techniques, the success is not guaranteed. Biophysical computations show that it depends on factors such as the fraction of time in which they are co-activated (Makarova et al. 2011), which is unknown *a priori* in experiments.

In general, gamma waves have an average duration roughly approaching that of the unitary synaptic inputs. Thus, their build-up into FPs requires highly synchronous activation of upstream neurons that is naturally provided by their grouping into functional assemblies (Cardin et al. 2009). Intense experimental and theoretical research that had not in account the spatial dimension has yielded different models to explain the emergence of FP gamma oscillations. These range from autocoherence emanating from self-organizing emergent properties of the network to resonant stochastic oscillators (Vida et al. 2006; Xing et al. 2012). The exploration of these models typically uses time-frequency analysis of FP time series (e.g. Burns et al. 2010), which implicitly assume high locality and single source origin of FPs. As appreciated in Fig. 5A and B, such neglects may have frustrated the conclusions.

## Final remarks

Despite having settled into the age of multi-site recording, the praxis and conceptual armor of many who study FPs is still strongly ingrained in choosing good electrode locations with the uncertain goal of recording spikes and FP activities elicited by the neuron population of interest. The expectation that these two activities that represent outputs and inputs are causally related contrasts with the abundant literature that seems to show partial and/or occasional relationship. Obviously, differences in their nature and mechanisms of generation account for the discrepant results (Murthy and Fetz 1996; Liu and Newsome 2006; Nauhaus et al. 2009; Okun et al. 2010; Ray and Maunsell 2011; Herreras 2016). But most important, the FP dynamics is settled by neurons upstream to those that generate the currents, and several populations typically converge on a common target where the synaptic currents blend and distort each other’s time course. We thus argue that interpreting the voltage fluctuations in raw FPs is hindered by neglecting their multisource origin. According to basic principles of field theory (Lorente de Nó 1947a; Plonsey 1964; Nunez and Srinivasan 2006) not only the time course does not reflect accurately the sources, but it varies in different sites. A good descriptor of FPs would be: one site, one blend, one waveform.

We are used to thinking that stereotypic temporal patterns like rhythms or short FP events originate from unique neural processes, but in most cases explored so far they are not, and the temporal fluctuations vary in different recording sites according to the relative strength of the potentials from each co-activated sources. Needless to say that recovering the time course of the sources requires prior disentanglement, for which multisite recordings and BSS algorithms have proven highly efficient (Makarov et al. 2010; Martín-Vázquez et al. 2013; Głąbska et al. 2014; Schomburg et al. 2014; Herreras et al. 2015). Importantly, the successive waves in common oscillatory rhythms such as the CA3-CA1 gamma represent distinct packages of information set by varying afferent cell assemblies, an information that is locked up in the waveform (Fernández-Ruiz et al. 2012b; Benito et al. 2016).

We have also emphasized that such spatial mixtures of potentials are not necessarily contributed only by co-localized sources arising from the same or nearby populations to which the electrodes are aimed. It is even more common that sources far from the recording electrodes contribute significantly (Whitmore and Lin 2016; Carmichael et al. 2017; Parabucki and Lampl 2017; Torres et al. 2019). The little attention given to distant sources is due to insufficient awareness of the relationship between the spatial geometry of sources and the rate of decay of associated potentials.

Formal exploration of FPs with single-site recordings may therefore require published data to be carefully re-assessed and/or re-interpreted. This affects but is not limited to frequency spectra, auto- and cross-correlations, CSD analysis, wavelet analysis, coherence analysis, machine-learning approaches for pattern seeking, and any quantification of the waveform parameters of spontaneous FPs that does not take into account their site-dependency and multisource nature. One must be aware that an important amount of noise may be introduced by undetected remote contributions. No doubt the levels of statistical significance will shift considerably after their removal, resulting in some relationships being unmasked and others found to be spurious. The unraveling of the pathway-specific time courses blurred in multisource LFPs may be particularly unexpected.

We tried to emphasize that the “contamination” of recordings by potentials from distant sources is an incomplete and misleading formulation. Volume conduction is not a problem that occasionally affects FPs but rather an inescapable condition of neural tissue that affects any FPs in the brain, including LFPs. Whether co-activated sources are distant or close, their potentials mix and should be treated similarly: disentangling. After source disentangling all time-frequency analyses mentioned above gain reliability and anatomical interpretability. FPs are widely accepted as biomarkers or correlates of behavior, and valued in biomedical disciplines oriented towards phenomenology or diagnosis. We advocate for a continued development of efficient algorithms for separation, and intensive benchmarking with synthetic spatiotemporal mixtures of sources in order to reinforce the usability and interpretability of FPs.

## Supplementary Material

video_1_bhac297

Video_2_bhac297

SitedependentCC_supp_video_legends_bhac297
