# Supplementary material for: Site-dependent shaping of field potential waveforms
Source: Cereb Cortex. 2022 Aug 16;33(7):3636–50. doi: 10.1093/cercor/bhac297 (PMC10068269; doi:10.1093/cercor/bhac297)
Supplement: SitedependentCC_supp_video_legends_bhac297 [file sitedependentcc_supp_video_legends_bhac297.docx]

**Video 1.** Variable spatial reach and mixing of field potentials in the brain. Three-dimensional representation of the running potentials elicited by cortical and hippocampal neuron sources of current. The spatial structure of the sources is shown in black and takes the form of curved sheets of current that represent the neuronal domains through which current enters or leaves the extracellular space. The changing concentric spheroids represent isopotential surfaces (blue and red are negative and positive values) and their greater or lesser extent reflects the amplitude of the local currents in the source populations. In the bottom row the activities modeled are: slow cortical waves (left), theta rhythm in the CA1 hippocampal region (middle), and alpha-gamma waves (right) in the dentate gyrus (DG). They each have distinct behavioral correlates, but they may also appear together. The upper plot combines all these. The model is based on finite-element methods using realistic dimensions, current densities and temporal dynamics (as in Torres et al. 2019). All the activities reflect patterned synaptic activation elicited by afferent pathways onto subcellular domains of the respective target populations. Note the spatially sliding activation of cortical and hippocampal waves, albeit with different coverage and velocity, which obey the different extent of coherent activation over the population and the mechanism of spread, respectively. Alpha-gamma waves are modeled as synchronous across the septo-temporal axis of the DG (but may not be). All the activities are dipolar and show an inhomogeneous spatial extent outside the respective sources. As expected, the addition of positive and negative potentials is particularly effective in distorting spatial features (compare the top display with any at the bottom). To get closer to the experimental recordings, all potentials are AC-filtered. Computed potentials have not been cut off at the boundaries of the brain.

**Video 2.** A homogeneous population harbors different source geometries depending on the portion of its dendritic arborization that is activated by one or another pathway. The video shows the LFPs generated along the strata of the CA1 pyramidal population upon input from several synaptic pathways (upper left scheme). The LFPs are represented as two-dimensional contour plots (lower left panel) inside the source population and as customary single-site recordings (e1-e7) obtained from a linear multielectrode probe (red and blue are positive and negative potentials, respectively). The dynamics of synaptic inputs is depicted in the running plot of the upper right box (each stroke marks the timing of an input: G1 and G3 are set excitatory and G3 is inhibitory). Some pathways afferent to a population promote significant buildup of FPs with characteristic spatial (depth) profiles, which are easily recognizable when activated alone (e.g. G1 in the first half of the run). In this epoch, the site-dependent FPs have proportional amplitude (positive or negative) inside the source population, and they keep identical temporal dynamics. However, the waveforms produced by this pathway become barely recognizable during the joint activation of several pathways (G2 and G3) in the same population (second half). The uneven addition of positive and negative potentials in different strata causes the site-dependent FP mixture to display different time-courses. The spatial coverage of the instantaneous potentials can be better appreciated by freezing the video at desired moments. The spatiotemporal summation of postsynaptic currents inside single cells underlies the site-dependent time-course of mesoscopic FPs, which are then unreliable even when they are generated by a single population and recorded in its vicinity. Timings of inputs are arbitrary. Modified from Herreras et al. 2015).
